# Connecting the Dots Between Inflammatory Bowel Disease and Metabolic Syndrome: A Focus on Gut-Derived Metabolites

**DOI:** 10.3390/nu12051434

**Published:** 2020-05-15

**Authors:** Andrea Verdugo-Meza, Jiayu Ye, Hansika Dadlani, Sanjoy Ghosh, Deanna L. Gibson

**Affiliations:** 1 Department of Biology, University of British Columbia, Okanagan campus, Kelowna, BC V6T 1Z4, Canada; averdugo@mail.ubc.ca (A.V.-M.); jiayu.ye@ubc.ca (J.Y.); hansikadadlani9@gmail.com (H.D.); 2 Department of Medicine, University of British Columbia, Okanagan campus, Kelowna, BC V1V 1V7, Canada

**Keywords:** immunometabolism, gut microbiome, microbiomics, insulin resistance, metabolism, inflammatory bowel disease

## Abstract

The role of the microbiome in health and disease has gained considerable attention and shed light on the etiology of complex diseases like inflammatory bowel disease (IBD) and metabolic syndrome (MetS). Since the microorganisms inhabiting the gut can confer either protective or harmful signals, understanding the functional network between the gut microbes and the host provides a comprehensive picture of health and disease status. In IBD, disruption of the gut barrier enhances microbe infiltration into the submucosae, which enhances the probability that gut-derived metabolites are translocated from the gut to the liver and pancreas. Considering inflammation and the gut microbiome can trigger intestinal barrier dysfunction, risk factors of metabolic diseases such as insulin resistance may have common roots with IBD. In this review, we focus on the overlap between IBD and MetS, and we explore the role of common metabolites in each disease in an attempt to connect a common origin, the gut microbiome and derived metabolites that affect the gut, liver and pancreas.

## 1. Introduction

**Figure 1:**
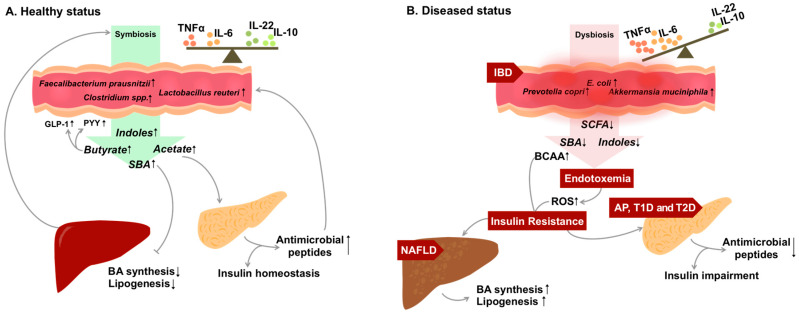
The host–microbial metabolites interplay in health and chronic inflammatory disorders. Gut microbes produce metabolites that act either at local tissue or remotely in the liver and pancreas (**A**). In inflammatory bowel disease (IBD), damage to intestinal permeability is associated with changes in microbiota composition (dysbiosis). Consequently, this disrupts the homeostasis of microbial-derived metabolites, leading to increased inflammatory potential locally and systematically (**B**). Ultimately, protective metabolites (SCFA, SBA and indoles) are decreased, whereas pre-disorder metabolites such as BCAA are increased. This further disrupts the energy metabolism by impairing the insulin signaling pathway and accumulating fat droplets in the liver, as a result of developing hepatic (NAFLD) and pancreatic disorders (AP, T1D and T2D). The production of two energy regulating hormones, GLP-1 and PYY, is triggered by the secretion of SCFA (butyrate). With decreased SCFA in disease state, production of GLP-1 and PYY is also limited, therefore worsening the disrupted energy metabolism. AP: acute pancreatitis; BA: bile acid; BCAA: branched-chain amino acid; GLP-1: glucagon-like peptide 1; NAFLD: non-alcoholic fatty liver disease; ROS: reactive oxygen species; PYY: peptide YY; SBA: secondary bile acid; SCFA: short-chain fatty acids; T1D: type 1 diabetes; T2D: type 2 diabetes.

Metabolic syndrome (MetS), or its consequence type 2 diabetes mellitus (T2DM), has been believed to be the direct result of a lack of exercise or caloric excess culminating in excess adiposity. While this remains true, we are observing an increased emphasis on the pervasive role of inflammation in multiple chronic health conditions, including inflammatory bowel disease (IBD) and nonalcoholic fatty liver disease (NAFLD), showing similar metabolic adaptations and insults connected to obesity and T2DM. This forces us to consider a common etiology of multiple chronic diseases while eliminating organ-specific problems as the root cause. For the sake of this review, we will consider the gut microbiome and its derived metabolites as a major common underlying driver of IBD and MetS. The purpose is to highlight the biochemical cross-talk between the gut microbiome and gut-derived metabolites associated with chronic inflammation to metabolic defects leading to liver and pancreas pathologies like NAFLD and diabetes. In particular, we focus on the current knowledge unraveling the mechanistic interaction between gut-derived metabolites in IBD and the collateral risk factors on promoting immuno-metabolic disorders like MetS. In this review, we propose that gut-derived metabolites shaped by the microbial ecosystem residing in the gut serve as a potential common origin connecting IBD, MetS and associated diseases like T2DM (Figure 1).

## 2. Commonalities between Inflammatory Bowel Disease and Metabolic Syndrome

Several parallels exist between MetS and IBD, both recognized as chronic life-long diseases. For two different clinically presented diseases to be related, one must consider its commonalities and co-existence. MetS refers to the cluster of risk factors such as obesity, insulin resistance, dyslipidemia and hypertension, leading to increased risk of detrimental metabolic diseases such as T2DM and cardiovascular disease [1]. Westernized countries like the United States report more than 30% MetS prevalence, and globally at least 25% of the population has MetS [2]. Another “Westernized” disease, IBD, is a group of disorders including ulcerative colitis (UC) and Crohn’s disease (CD), characterized by chronic and relapsing inflammation in the gastrointestinal tract [3,4]. North America and Europe have the highest IBD incidence worldwide, with a prevalence of 0.5%, extrapolated to 1.5 million diagnosed IBD patients in North America and 2 million in Europe [5,6]. Despite IBD not being at the top of the list of global killers, unlike MetS and associated diseases [7], the number of deaths and burden may have a comparable impact in the future as the prevalence continues to rise (85.1% increase from 1990 to 2017) [8]. Although linked to genetic susceptibility, heritability of disease remains weak for MetS and IBD (<25% for MetS, <15% for UC and <50% for CD) [1,6]. Therefore, it has been implied that an additional ‘environmental trigger’ is a must, but this remains undefined. As an example, a cohort study in Canada looked at the incidence of IBD between immigrants and the domestic population. Offspring of immigrants from Asia, of which typically have lower incidence of IBD, showed significantly increased IBD cases, compared to that of children of non-immigrants [9]. However, immigrants who came to Canada at a later age did not have an increased incidence of IBD. Although the exact mechanism remains unknown, environmental change at an earlier age is most likely considered a major player in the origin of IBD. Environmental factors are well known to be a group of driving factors in increased incidence and risk of MetS [2].

One of the most studied shared mechanisms of chronic diseases has been inflammation. Jurjus et al. (2016) extensively reviewed the connection between the inflammatory loops in both IBD and T2DM [10]. The authors highlighted the preponderant role of macrophages and their pro-inflammatory mediators in causing IBD. Similar mechanisms have also been proposed for T2DM. Common mechanisms include increased production of TNF-α, IL-6 and reactive oxygen species (ROS) [10,11]. Similar to T2DM, this trend is also associated with the risk of obesity. Alteration of leptin [12], obesity-correlated hormones, dietary intervention [13] and bariatric surgery [14] all attenuate symptomatic obesity through lowering these pro-inflammatory cytokines in circulation. Blocking inflammation via specific agents show commonalities in their effects on both MetS and IBD, at least in some studies. Anti-TNF-α agents such as infliximab have been approved for adults and children with CD in Europe [15,16]. Infliximab therapy showed an improvement in insulin sensitivity in patients with rheumatoid arthritis, a chronic inflammatory disorder [17,18]. However, while infliximab reduced disease activity in CD patients, insulin sensitivity was not altered [19,20]. Given the various pro-inflammatory molecules involved in impairment of insulin signaling as well as CD, it is highly unlikely that inhibition of one single pro-inflammatory molecule could improve the insulin-signaling cascade and highlights the inability of current therapy in muting the inflammation cascade in its entirety. Overall, it is clear that pro-inflammatory factors lead a significant role in the development of these diseases. The question remains as to what is the origin of this dysregulated chronic inflammation?

Evidence for a common origin is stronger when IBD and MetS are considered as co-morbid conditions [15]. Although in the past IBD patients were considered lean or underweight due to malabsorption of nutrients, more studies are reporting a considerable number of IBD patients being obese and overweight following treatment [21,22,23]. A nationwide population-based cohort in Denmark found that out of the 65,180 patients diagnosed with IBD, 50% showed an increase in risk of T2DM compared with the general population [24]. Another nationwide population-based study in South Korea showed a similar result, where CD patients from 8,070 IBD patients were shown to have a higher risk of developing T2DM. Interestingly, this observation was primarily driven by younger individuals with IBD [25]. Similarly, a small study from a German cohort demonstrated higher insulin levels in CD patients when compared to controls (*p <* 0.01) [26]. Moreover, up to 32% of IBD patients show NAFLD [27] compared to 24% in the normal population [18]. It has been observed there is an increased risk of developing atherosclerosis and later progression to cardiovascular disease in IBD [16,20,28,29]. Thus, recognition of NAFLD and atherosclerosis, which are well-recognized aspects of the MetS spectrum in IBD patients, indicates a strong co-morbid relationship between IBD and MetS. Therefore, it appears that such pro-inflammatory factors lead a significant role in the development of diseases, but the question remains as to what the origin of these diseases is.

## 3. Dysbiosis as a Common Feature of IBD and MetS

In line with the clinical observations that IBD and metabolic diseases may be comorbid, one mechanism by which these diseases might be connected, and as a common origin, is through the gut microbiome, or rather an alteration of the normal healthy microbiome referred to as dysbiosis. In the last decade, gut microbial dysbiosis has emerged as one of the novel mechanisms contributing to diabetes via increased intestinal permeability leading to systemic chronic inflammation. The compromised gut barrier function allows close contact of gut bacteria with gut epithelium and ultimately enhances infiltration of immune cells, expression of pro-inflammatory cytokines [30] and oxidative stress [31], leading to free lipopolysaccharide (LPS) entering into circulation causing endotoxemia [32] or high levels of endotoxins such as LPS in blood plasma. Low subclinical inflammation can lead to the onset and progression to T2DM by developing insulin resistance. 

Fecal transplantation can either exacerbate or ameliorate IBD and metabolic diseases. Fecal transplantation experiments in mice can influence colitis susceptibility patterns [33,34]. Some models of colitis entirely depend on the presence of gut microbiota including IL-10^−/−^ model of colitis [35,36]. Similarly, obesity is a phenotype controlled by the mere presence or absence of obese-associated gut microbiota revealed in class fecal transplant experiments in germ-free mice using both human and rodent gut microbiota [37]. Furthermore, glucose tolerance [33] and insulin resistance [38] are similarly controlled by the mere presence of the gut microbiome, evident by the switch in phenotypes via fecal transplantation experiments. These experiments suggest the gut microbiome is a causal factor in IBD, obesity and T2DM, although it is not clear if individual taxa or overlapping functional metabolites are the major players in disease phenotypes. 

The taxonomical changes of the gut microbiota and the development of metabolic diseases have been reviewed by many [34,39,40] and will only be briefly touched upon here. The pathological change in gut environment in both IBD and MetS affects the symbiosis between the gut and microbiota, supporting a switch to species able to thrive in the highly inflamed and oxidized gut with a suppression in more strictly anaerobic microbes. What is curious is that both dysbiotic states described for IBD and MetS and their associated outcomes on species distribution are similar. Decreased richness is a common feature between IBD, obesity, insulin resistance, T2DM and NAFLD; while an increase in Proteobacteria and decrease in Firmicutes is a common trait for IBD [41], insulin resistance [42], T2DM [43], NAFLD [44] and atherosclerosis [45]. Specific examples overlapping between IBD and metabolic disorders include the decreased presence of particular *Clostridium* spp, *Faecalibacterium* and *Roseburia*, and increased representation by *Enterobacteriaceace*, *Blautia* and *Rumminococcus* [3,41,43,45,46,47,48,49,50]. Indeed, certain microbes like *Clostridium* spp. exert anti-inflammatory functions, promoting regulatory T cells through TGF-β release [32], an overlapping pathway in both IBD and MetS. Although this area is still under active research, increase of *Escherichia coli* and *Fusobecaterium nucleatum* species, and variations in *Akkermensia muciniphila,* are good examples of commonalities in IBD and MetS dysbiosis. In summary, gut microbial dysbiosis is similarly associated with the chronic immune nature of IBD and MetS. 

### High-Fat Diets and Dysbiosis

The most pervasive environmental influence that could provide a ‘trigger’ to susceptible genetic patterns is the food we eat. Diet, known to cause dysbiosis, is one of the environmental triggers for the onset of IBD, MetS and associated diseases. Several studies have shown that while dietary fiber promotes the growth of bacteria that have the ability to decrease or resolve inflammation [51,52], high-fat diets tend to promote low-grade inflammation that alters gut barrier function [53]. It is well established that a high-fat diet exacerbates MetS or diabetes. High levels of unsaturated fatty acids can promote production of oxidized lipid metabolites that trigger systemic inflammation [54]. High-fat diets cause intestinal inflammation as well obesity, adiposity and insulin resistance [55]. Considerable evidence suggests that high-fat diets contribute to endotoxemia [56,57]. This explains the similar increase of LPS in blood observed in patients with diabetes and obesity [55]. A high-fat diet induced colonic inflammation and increased nitric oxide synthase (iNOS) expression during LPS-induced inflammation via toll like receptor (TLR)-4 signaling [10,11,58,59]. A similar increase in TLR-4 was reported for intestinal and colonic samples of UC and CD patients [60]. Elevated TNF-α, IL-6 and iNOS are important molecular links between inflammation and insulin sensitivity. TNF-α inhibits insulin receptor substrate-1 (IRS-1), one of the first nodes of the insulin signaling pathway, by phosphorylating it at serine residues, thus inhibiting signaling downstream of insulin receptor [61,62,63]. IL-6 decreases tyrosine phosphorylation of IRS-1, and hepatic cells when treated with IL-6 experienced complete loss of Akt activation [64]. Furthermore, iNOS promotes deactivation of downstream nodes of insulin signaling pathways including Akt, IRS-1 and IR, inducing insulin resistance [17,18,65,66]. Inhibitors of iNOS improve hepatic insulin resistance in genetically obese mice, demonstrating the detrimental role of iNOS on impairing insulin signaling [67]. A study revealing high iNOS expression in pancreatic islets from diabetic individuals supported this proposition [68], which is also observed in active IBD patients [69]. 

Not all high-fat diets are similar in their ability to cause gut dysfunction, and diets rich in n-6 and polyunsaturated fats drive compromised gut barrier function resulting in exacerbated colitis [70,71]. In previous studies, we have shown that gut health influences the immune-metabolic system, and that the gut microbiome is adversely affected with corn oil diets rich in omega-6 polyunsaturated fatty acids [71,72,73,74]. In a recent study, we demonstrated that consumption of corn oil led to metabolic dysfunction and insulin resistance in mice compared to the beneficial olive oil [75]. In parallel, we have shown that this exact diet also promotes intestinal barrier dysfunction and systemic chronic inflammation associated with colitis [73]. We have demonstrated that feeding a corn oil diet leads to a pro-oxidative state, where pro-inflammatory microbes flourish, causing barrier dysfunction, oxidative stress, inflammation and insulin resistance [71,74,76,77]. Overall, high-fat diets rich in omega-6 polyunsaturated fatty acids may be an environmental factor that triggers dysbiosis common to IBD and MetS. 

## 4. Consequences of Dysbiosis: Microbiome vs. Metabolome

While current evidence suggests that the gut microbiome does dictate the origin and development of IBD, MetS and related metabolic diseases, the question remains to as to how. There are two plausible broad mechanisms as to how the gut microbiome can affect systemic events. Firstly, the gut being the gateway to the entry of food as well as airborne and water borne microbes and other substances serves as the ‘gatekeeper’, alarming the system towards either a placid or an agitated response. This, in other words, is the immune-mediated pathway that has been well studied especially in the case of colitis and T2DM. In this scenario, the gut microbes themselves or toxins enter through a ‘leaky’ gut into the lymphatic or the blood evoking proximal (from the gut wall) or distal (various organs) responses via cytokines and chemokines. In support of this, several models of colitis do show a preponderance to metabolic defects (summarized in Table 1). Indeed, chemical (DSS and TNBS) [78,79] and genetically susceptible (IL-10^−/−^, Mdr1a^−/−^ and Muc2^−/−^) [80,81,82] rodent models of IBD display gut barrier dysfunction in which an increase in pro-inflammatory cytokines and oxidative stress are part of the phenotype. In fact, the disturbances in IL-1β IL-6, TNF-α or ROS are described in the colon as well as in the liver and pancreas. For example, in the chemical DSS model, increase of those pro-inflammatory cytokines and oxidative stress evokes colon signals that reflect in metabolic defects, including decreased plasma insulin and ROS in the pancreas [83], and higher TLR4, TLR9, MCP-1 and TNF-α expression in the liver, resulting in liver fibrosis [78,84]. Further, in a model of dyslipidemia with higher circulating cholesterol, ApoE^−/−^ mice given DSS not only resulted in the expected barrier dysfunction but also glucose tolerance impairment, liver fibrosis and exacerbated aortic plaques [85]. For the TNBS model, intestinal inflammation leads to endotoxemia, liver inflammation and increased hepatic triglycerides and cholesterol, progressing to liver damage [79,86,87]. This outcome worsened when rodents were exposed to a ‘second hit’ from high-fat diets augmenting fat deposition in the colon and liver. This pathologic cross-talk includes adipose tissue, which shows dramatic increases in inflammatory markers IL-1β, IL-6, MCP-1 and TNF-α, as well as decreased anti-inflammatory adiponectin [88]. Spontaneous colitis models display low-grade inflammation and low glucose levels (Muc2^−/−^) [82] but increased insulin levels (IL-10^−/−^) [80]. During high-fat diet exposure in the Mdr1a^−/−^ model of colitis, disease worsens in the colon and liver [81], equivalent to the proximal and distal response proposed earlier. 

In the second scenario, the gut microbiome could act as a ‘facilitator’ enhancing and protecting against metabolic dysfunction in the liver and pancreas or instead expediting pathology. The surface area of the human gut is approximately 300 m^2^ and is a hotbed for production of diverse metabolites from various dietary substrates like proteins and digestible and indigestible carbohydrates like fibers. The nature of the gut is emphasized by the fact that each area of the gut is specialized with specific enterocytes with varying metabolic functions. As an example, while the small intestine is primarily involved in the digestion of food and absorption of nutrients, the large intestine is primarily involved in water absorption and elimination of waste. As diverse enzymatic reactions need specific pH ranges, the gut also demonstrates variations across its length, which might indicate specific metabolite production across these areas. Duodenal pH is raised to around 6, from 2 in the stomach, with the help of bicarbonate buffers from the pancreas. The pH keeps rising and eventually reaches 7.4 in the terminal ileum. Subsequently, the pH drops to 5.7 in the cecum and ends at 6.7 in the rectum [89]. While the host-driven pH ranges might dictate a niche of various microbes, differentiating species among a certain taxa can be challenging due to the complexity of the intestinal environment [90]. Acidic environments (pH~5.5) promote fiber fermentation and butyrate, a short-chain fatty acid (SCFA) producing species. In addition, a low pH environment inhibits the activity and secretion of proteolytic enzymes, therefore slowing down the protein/amino acid metabolism in the gut [91]. Intraluminal pH in patients with IBD (UC and CD) is higher than that in healthy individuals, which inhibits colonization of butyrate-producing bacteria and slows down fermentation of fibers [92,93]. This, in turn, promotes a widely different microbial niche that may lead to chronic diseases. In this regard, although UC and CD have distinct inflammatory responses and metabolic pathways, they both have increased Firmicutes (as described for T2DM) [41]. However, Bacteroidetes have inconsistent performance in UC and CD [94]. Clinical studies indicate that the ratio of Firmicutes to Bacteroidetes can be used as the clinical marker of IBD incidence. Considering the shift of the intestinal environment results in bacterial alteration, the output of metabolites may, therefore, be affected as well. 

Despite informative metagenomic analysis, the science of correlating pseudo-pathways with the actual metabolic signature is still in its infancy. In addition, differentiating between host and microbial metabolites is challenging. As an example, there is no chemical difference in NADPH or butyrate produced by microbes or the host cell, both of which need the molecule as a reducing equivalent. Thus, as fundamental molecules of life are common, it is even more probable that multiple microbes instead of a single microorganism drive specific metabolic signatures. To support this, a plethora of studies that replicate disease phenotypes from humans into mice after receiving fecal microbiome transplants from patients [34] have shown the need of the entire microbiome, including many taxa and their metabolites, to drive the disease. As it is difficult to relate an entire disease phenotype to only one microbe, studying a particular metabolite, albeit produced by multiple taxa, might be more suitable to decipher the disease process. 

Moving away from microbe-centric causality of chronic diseases to a metabolite-specific pathway sheds light on the enigma of the biome, as metabolites produced by a single species, or multiple microbes, might signal the harbinger of chronic diseases. There are several gut-derived metabolites shared between IBD and MetS (Table 2). As examples, Gram-negative bacterial products such as LPS, long associated with sepsis, are now being studied in NAFLD [105]. Novel dietary choline-derived microbial metabolites like trimethylamine N-oxide (TMAO) are being investigated as a pathological trigger in cardiovascular disease, a direct consequence of MetS [58]. As analysis and discovery of metabolites becomes more sophisticated with greater resolving power and clarity through advances in analytical instrumentations like mass spectrometry, inter-connectivity of diverse chronic diseases is becoming clearer. Such discoveries are also being facilitated by the use of germ-free mice vs. conventionally raised mice. Now we can discern how the microbiota influences host-circulating metabolites [106], feces metabolites [107] and certainly gut metabolites [108]. These studies help us understand which metabolites are host derived and which are microbial derived. For example, the amino acids tryptophan, phenylalanine and tyrosine are present at higher concentrations in the serum of germ-free mice, whilst their metabolites serotonin and phenylacetylglycine are increased in conventional mice [106].

## 5. Gut-derived Metabolites: Assessing biochemical commonality between IBD and MetS

Much research needs to be done to understand the biochemical parallels between IBD and MetS metabolic signatures in any depth. The disturbed environmental niche in the colon also marks a higher incidence of IBD and obesity [42]. Obesity correlates to many metabolic disorders, but typically marks the imbalanced energy metabolism, especially lipid and glucose. In this section, we will focus on known metabolites that shift in their abundance, which could provide a starting point in further understanding the ‘gatekeeper’ or ‘facilitator’ role of dysbiosis in these diseases (Table 2). The connection between the dynamic metabolites secreted by the IBD gut microbiota and the development of MetS can be addressed by studying their impact on extra-intestinal tissues. Comprehensive reviews of the liver–gut axis [109] are some examples of the extensive literature explaining the most recent connections found between host physiology and microbiota.

### 5.1. Bile Acid

The liver is often the entry point of various oral substances systemically, including nutrients, which is connected anatomically to the gut through the portal vein representing the enterohepatic circulation. Bile acid metabolism has been widely studied for decades, with the primary bile acid (classic pathway) synthesized in the liver, whereas the secondary bile acid (SBA; alternative pathway) is produced in the large intestine by bacteria [184]. Around 95% of the primary bile acids are recycled back to liver through hepatic portal circulation. The remaining 5% primary bile acids move down to the distal ileum and are absorbed by enterocytes for further processing by the gut microbiome. Numerous clinical studies have disclosed the positive relations between SBA and gut health. Both malabsorption of primary bile acid and hepatic disorders lead to disrupted SBA production as a result of dysbiosis. However, bile acid metabolism and the bacterial community are mutually regulated. When compared to healthy individuals, IBD patients had lowered SBA profile, but higher primary [185] and sulfated bile acids in feces [186]. This observation is also accompanied by a significantly decreased ratio of *Faecalibacterium prausnitzii* and *E. coli,* which has been widely considered to be the biomarker of dysbiosis. 

Step one of SBA metabolism is deconjugation through bile salt hydrolase (BSH). Of the microbial community, Firmicutes is believed to have the most abundant and active form of BSH enzymes, followed by Bacteroidetes and Actinobacteria. Firmicutes and Actinobacteria are able to degrade all types of primary bile acids, whereas Bacteroidetes are only active regarding tauro conjugated bile acids [187]. Gut dysbiosis impairs the microbial community, which significantly reduces the capacity of this BA modification before entering into the large intestine. Efforts to reshape the bacterial community of IBD patients by introducing *Lactobacillus reuteri* (NCIMB 30242) has been attempted [185]. *Lactobacillus reuteri* is a known strain that equips bacteria-bearing BSH, which is essential to deconjugate primary bile acid. Nine-week administration of *Lactobacillus reuteri* improved SBA production of IBD patients, compared to the placebo group [185]. Unconjugated bile acids are passively reabsorbed to synthesize SBA in the large intestine. The following transformation and desulfation are processed by hydroxysteroid dehydrogenases (HSDs), which are largely produced by *Clostridium* genera. In other words, bacterial teamwork deconjugated and transformed primary bile acid to SBA more efficiently [162].

One other way how bile acid metabolism has shown to affect both IBD and metabolic syndrome is through farnesoid X receptor (FXR), which is present in both the liver and the intestine. FXR regulates glucose sensitivity, hepatic lipid homeostasis and also bile acid [188,189]. Primary bile acid binds to hepatic FXR, stimulating the downstream response of fibroblast growth factor 19 (FGF19). FGF19 and FXR co-regulate the homeostasis of primary bile acid production. FGF19 inhibits the synthesis of primary bile acid from cholesterol in the liver. Activated FXR is shown to inhibit lipogenesis through regulating the expression of SREBP1. Deletion of FXR in mice altered microbiome composition, with dramatically decreased Firmicutes in particular. Proteobacteria, instead, are dominant in the gut [190]. This interrupted microbiome community significantly affected the production of SBA by blocking production of Firmicutes derived BSH. As a result, FXR^−/−^ mice had decreased weight gain but developed hepatic inflammatory diseases such as NAFLDs and steatosis [191]. Antibiotics attenuate hepatic inflammatory responses by inhibiting Proteobacteria colonization in FXR^−/−^ mice exposed to a normal chow diet [190]. Similarly, FXR agonist (INT-747) reversed and attenuated symptoms of IBD in a chemical-induced colitis mice model. Increased FXR expression alleviated mice colitis by suppressing inflammatory responses with decreased expression of pro-inflammatory markers such as IL-6 and TNFα [166]. Curiously, compared to healthy individuals, patients with IBD and other metabolic disorders such as diabetes [192] accumulate bile acid and have less active bile acid detoxifying activity by inhibiting the FXR-associated energy metabolism [192,193]. Patients with metabolic disorders such as diabetes, hepatic diseases and atherosclerosis all indicate decreased FXR expression in the liver and gut [194]. FXR is the key to ensure the activity of mitochondrial function by reducing oxidative stress and provoking the expression of AMPK and PPAR-γ [195,196]. Overall, FXR plays as the core energy metabolic factor to regulate systemic energy homeostasis. Its activation stimulates FGF19 in the intestine, which regulates the bile acid pool by controlling bile acid synthesis in liver.

### 5.2. Short-Chain Fatty Acids

Short-chain fatty acids (SCFAs) are fatty acids with a carbon length of 10 or less. Straight-chain SCFAs are derived from indigestible carbohydrates, amino acids and host-derived glycoproteins, whereas branched-chain amino acids are the source of generating branched-chain SCFAs [197]. The most abundant SCFAs include acetic acid (C2:0) followed by propionic acid (C3:0) and butyric acid (C4:0). Various carbon-chain lengths determine the diversity of metabolic pathways that are mediated by different genera of intestinal bacteria. It is believed that *Bifidobacterium* spp. primarily produce acetate, whereas *Akkermansia muciniphila* can produce both acetate and propionate [198]. However, the list of bacteria that can metabolize butyrate is much longer, with most of the species belonging to phylum Firmicutes [198]. It is worth noting that other SCFAs such as valeric acid and caproic acid are also closely associated with chronic disorders [199,200]. For example, valeric acid, together with butyric acid, showed to protect against colitis and CVD by suppressing histone deacetylase, which is a risk factor for inducing IBD and MetS [201]. Intestinal SCFAs can enter enterohepatic circulation to regulate lipid and glucose homeostasis in gene and hormone manners. Glucagon-like peptide (GLP-1) and peptide YY (PYY) are two major nutrient-stimulated hormones that are released from enteroendocrine L cell in the colon, where SCFA production is at a higher level [202,203]. The production of GLP-1 and PYY is driven by SCFA-associated G coupling protein, which will be discussed later. It has been well studied that GLP-1 and PYY both control energy intake. Patients with metabolic syndrome such as diabetes and heart diseases typically have reduced GLP-1 and PYY in circulation [204]. One study showed that SCFA and GLP-1 levels remained low in hyperinsulinemic patients. These patients were intervened with a high-fiber diet for one year to try to increase SCFA production. After one year, dietary intervention improved insulin sensitivity, with elevated SCFA production and circulatory GLP-1, compared to the low-fiber diet group [205]. SCFA supplementation (butyrate) also showed to improve NAFLD and insulin resistance [156].

High-fat diet is another risk factor for provoking IBD and MetS, as we discussed earlier. Mice exposed to either high-fat or high-sugar diets have reduced SCFA [206]. Our group [75] and many other groups [207] have demonstrated the role of HFD on stimulating metabolic disorders such as NAFLD, CVD and diabetes. HFD disrupts the shape of intestinal bacteria, as a result of reducing SCFA production and increasing gut permeability [206,208]. Simultaneously, HFD also interrupts energy metabolism, which induces systemic inflammation, insulin resistance, dyslipidemia as well as the corresponding MetS. In summary, regardless of disease type, maintaining SCFA and its derived hormone are the central role in ensuring energy homeostasis, therefore reducing the risk of MetS and IBD.

### 5.3. G protein-Coupled Receptors

Many studies have demonstrated that SCFAs improve glucose and lipid homeostasis via regulating G protein-coupled receptors (GPCRs). GPCRs are the receptors that directly regulate SCFA metabolism such as the production of GLP-1. Two major GPCRs, GPCR40 and GPCR43, sense the concentration of colonic SCFAs, which further stimulate the release of GLP-1 and PYY from the L cells. GLP-1 and PYY are hormones that control appetite. GLP-1 stimulates the production of insulin in starvation, which has been well-studied as an anti-diabetic target. Patients with UC have decreased sensitivity of glucose with significantly reduced GLP-1 production [209]. Hence, GLP-1 agonist has long been researched as the therapeutic target on attenuating metabolic disorders such as diabetes. It is the FDA approved medication for patients with T2DM to reduce blood pressure, lipid accumulation and improve insulin resistance [210,211]. Similar to glucose, dietary fatty acid metabolism is also highly determined by the production of intestinal SCFAs. Many studies have reported that acetate and butyrate control hepatic lipogenesis [35]. However, patients with NAFLD and NASH have higher production of acetate and propionate, but decreased butyrate, compared to healthy individuals [120]. This is believed to be the result of modification of bacterial diversity. Therefore, restoring microbial community may help to re-establish intestinal homeostasis.

### 5.4. Trimethylamine N-Oxide

Trimethylamine N-oxide (TMAO) was one of the first gut-bacterial derived metabolites recognized to be involved in the pathophysiology of cardiovascular disease (CVD). Gut bacteria catabolize choline and L-carnitine into trimethylamine (TMA), which is further oxidized in the liver to trimethylamine-N-oxide [176]. The seminal study by Wang and collaborators (2011) demonstrated how TMAO plasma levels significantly correlated with CVD (three independent studies with more than 2000 samples from different subjects, *p* < 0.01) [58]. More importantly, the study went from correlation to causation when mice fed phosphatidylcholine (source of choline and precursor of TMA) displayed increased levels of TMAO in an obligate bacterial-dependent manner and with subsequent development of atherosclerotic lesions [58]. This approach was also used for determining L-carnitine bacterial metabolism as a source of TMAO and, hence, another trigger of CVD [176]. Interestingly, high levels of TMA/TMAO have been found in IBD patients [174] and in the animal models of colitis DSS and IL-10^−/−^ [102]. This may be enhanced by the increased levels of carnitine associated with the oxidized environment of gut in IBD [212]. Conversely, TMAO levels appear to be lower in the plasma of IL-10^−/−^ mice [103], which can reflect either liver damage (necessary for TMA oxidation) or the dependence of certain interaction between host and gut bacteria, not present in the experiment reported. As summarized in Table 2, TMAO also correlates with glycemic response impairment [182] and NAFLD [183].

### 5.5. Tryptophan Metabolites

Tryptophan is an essential amino acid, being the only with an indole structure (a benzene ring fused to a pyrrole ring) [213]. It is acquired from the diet, and it is mainly absorbed in the small intestine, but still, a fraction of the amino acid reaches the colon where is catabolized to indole metabolites by the gut bacteria [213]. Tryptophan has attracted attention in recent studies due to its correlation with disease status in animals and humans. High concentration of tryptophan has been found in serum samples from rodent models of IBD [97], as well as in feces from patients with CD [214]. This association could be related to dysbiosis, since bacteria aid in tryptophan transformation into important metabolites such as serotonin. However, there is more literature related to the protective role of tryptophan on IBD development, where even oral administration of tryptophan reduced disease severity in piglets and mice [131,132]. In fact, a large cohort study in UC and CD patients revealed a negative correlation between serum tryptophan levels and disease activity, reporting that responders to Infliximab show higher levels of tryptophan when compared to controls [215,216]. However, these last two studies work in different samples (feces for the former and serum from the later). Whilst a more recent study positively correlated increased kynurenic acid (tryptophan derived metabolite) with IBD severity [217], meaning that the low concentration of serum tryptophan responds to an increased gut metabolism along with inadequate absorption of tryptophan by epithelial cells. 

More conclusive knowledge about the role of tryptophan metabolites in IBD relies on indole-propionic acid (IPA). This metabolite is produced by many gut bacterial species [218], and it has been associated with benefits in both IBD and MetS. Serum levels of IPA have been reported to correlate with IBD and MetS. For IBD, a study in 35 patients with UC found a 60% reduction of IPA in serum from patients with active UC when compared to healthy controls (*p* < 0.05) [133]. Regarding MetS, a study in 1018 patients reported strong, negative association of IPA levels with arterial stiffness, fasting glucose, insulin resistance and visceral fat (*p* < 0.05) [218]. Furthermore, indole levels also correlated negatively with liver fat deposition [219]. 

Beyond these associations, experimental studies with tryptophan metabolites have shown to enhance barrier function through goblet cell stimulation [220,221], acting on L cells and promoting the release of the incretin GLP-1 [218,222], exerting antimicrobial effects, and further inflammatory attenuation through activation of aryl hydrocarbon receptor (AhR) [220,223]. AhR is a transcription factor present in immune cells, and its stimulation via indoles leads to differentiation of CD4+ T cells into regulatory T cells (Treg), which exert anti-inflammatory functions [213], in this scenario through IL-22 secretion. Indeed, the administration of indoles in mice exposed to HFD as model of NAFLD protected them from liver damage and alleviated glucose parameters [219,224]. In rats, a single exposure to indoles improved plasmatic glucose and insulin levels [138]. Furthermore, two independent studies were able to describe the protective role of microbial-derived indoles in pre-clinical and clinical colitis and MetS [133,222]. Lastly, the protective role of indole metabolites is closely related to the SCFA butyrate, since the increase of IL-22 promotes butyrate-producing bacteria [220], and butyrate has been proposed to act as an AhR ligand too [225].

### 5.6. Branched-Chain Amino Acids

Another important set of metabolites connecting the gut microbiome with IBD and MetS is branched-chain amino acids (BCAAs), which are amino acids that have an aliphatic side-chain with a branch including leucine, isoleucine, valine and 2-aminoisobutyric acid. Interestingly, a study in pigs fed inulin, a non-digestible carbohydrate, improved cholesterol and glucose levels. Also, metabolomics analysis revealed significantly lower plasma levels of BCAAs, indicating a positive correlation with increased cecal beta-diversity [226]. In T2DM patients, a short-term dietary reduction of BCAAs modified the fecal microbiome, resulting in enriched Bacteroidetes and decreased Firmicutes [227]. This finding may correlate with changes in amino acid fermentative microbe genera such *Clostridium*, which disturbances have been reported for both T2DM [228] and IBD [105]. Analysis of normal, inflamed and dysplastic human colon tissue revealed differences related to BCAA in inflamed tissue [229]. In another study comprising fecal samples from pediatric patients recently diagnosed with IBD, differences in BCAAs (only valine and leucine), aromatic amino acids (phenylalanine and tryptophan), serine and histidine were significantly increased [230]. These clinical findings could be related to the importance of BCAAs in gut barrier integrity, since an increase in BCAA metabolism pathway (specifically leucine and valine enriched levels) has been associated with increased expression of mucins and tight junctions proteins in piglets [231]. Trying to merge these findings with MetS seems contradictory, since increased levels of circulating BCAAs are correlated to insulin resistance. In fact, patients undergoing bariatric surgery, which improves insulin resistance, show decreased circulating levels of BCAAs [232]. However, when BCAAs are administered to rats, they need to be on HFD in order to reproduce the insulin resistance phenotype [123]. An explanation to this lies in the fact that BCAAs can interfere with insulin signaling via phosphorylation of IRS1 [233]. Serine phosphorylation of IRS1 can minimize its activity and weaken insulin signal transduction [234]. Taken together, BCAA metabolism seems to be protective for IBD whilst is strongly associated with insulin resistance. The connection between these contradictory functions may be related to the inflamed gut, trying to sustain the gut barrier and meanwhile causing the oxidative stress behind insulin resistance onset.

## 6. Insulin Synthesis and Action

One of the cornerstones of MetS and the diabetic state is deficient insulin action, which arises due to a lack of insulin itself (type 1 and late type 2) or its effectiveness (early type 2). The most studied role of the gut microbiome is the “leaky gut” hypothesis. This suggests that microbial dysbiosis, a common finding in MetS [235], mediates a disrupted intestinal barrier function, which can lead to the translocation of pathogens and can eventually result in inflammation and insulin resistance. Gut dysfunction allows leakage of LPS into the circulatory system and leads to a chronic state of inflammation and endotoxemia [236], which results in impaired insulin secretion in pancreatic β-cells [237], pancreatitis and pancreatic cancer [238]. 

As both of these types of diabetes lead to glucose insensitivity, many metabolic signatures are similar as well as dissimilar [239]. The first factor that connects the gut to insulin secretion is the gut-derived metabolite-driven secretion of regulatory hormones like GLP-1 and gastric inhibitory peptide [135], collectively known as incretins [240]. An interesting study demonstrated that when germ-free mice were administered microbes from two different strains of mice showing varying insulin responses to glucose, the insulin release patterns of these two strains were transferred to the germ-free mice, signifying the critical role of the microbiome in insulin release [241]. Besides release, insulin synthesis is also influenced by microbial metabolism. As an example, increased acetate production induced by a HFD modulated the microbiome and promoted increased glucose stimulated insulin secretion in rodents [242]. Also, the production of cathelicidin-related antimicrobial peptide (CRAMP), a factor that protects β-cells of non-obese diabetic mice (NOD) from autoimmune diabetes and decreases expression of pro-inflammatory cytokines [243], is regulated by microbial metabolism. 

Acute pancreatitis represents inflammation of the pancreas that often leads to β-cell failure and precedes the development of type 1 mellitus (T1DM) and T2DM. A multihospital clinical trial revealed drastically different gut microbiomes in both mild and severe acute pancreatitis patients [244]. In experimental models, such findings are confirmed, as decreased microbiome diversity is noted in surgically induced acute necrotizing pancreatitis as compared to sham operated rats [245]. Acute pancreatitis has also been associated with lower diversity of the gut microbiome with an increase in Bacteroidetes and fewer Firmicutes in another study [246]. A dysfunctional gut barrier can propagate acute pancreatitis further as well. In this regard, patients with acute pancreatitis have higher plasma endotoxin and pro-inflammatory cytokines such as TNFα, IL-6, IL-10 and IL-8, along with increased intestinal permeability as compared to healthy volunteers, further strengthening results observed in murine models [247]. 

Insulin signaling in MetS can also be altered with dysbiosis. Heightened circulating LPS induces iNOS expression which promotes S-nitrosylation of Akt, IRS-1 and insulin resistance, deactivating nodes crucial to insulin signaling [65,66]. A study conducted on isolated human pancreatic islets from diabetic subjects and non-diabetics revealed significantly higher iNOS expression in the former [68]. Similarly, active IBD patients and healthy controls recapitulated this with higher iNOS expression in the former group [69]. Parallel increased iNOS expression has also been reported in rat models of IBD [248], pancreatic cancer [249] and acute pancreatitis [250,251]. Thus, it is obvious that enhanced iNOS expression represents a common critical point in the development of both IBD and MetS. 

Along with T2DM, microbiome studies in T1DM are constantly emerging. Clinically, T1DM has been associated with dysbiosis in children [252] as well as excessive antibiotic exposure in infants, which disrupts gut microbes [253]. A study done in infants concluded that communal diversity decreases and inflammatory metabolites and pathways increase even before the onset of T1DM [254]. In animals, antibiotic administration to NOD mice early in ontogeny during fetal and postnatal life increased incidence and progression of T1DM along with disruption of gut microbiota composition [255]. This outcome is somewhat expected, since the microbiome is involved in shaping and maintaining a balanced immune response since early in life [41]. In another study, fecal oral transplantation of NOD microbiome, a diabetogenic microbiome accelerated insulitis in non-obese resistant (NOR) mice, and antibiotic treatment further increased T1DM incidence in NOD mice. Antibiotic-induced diabetes was not only associated with microbial dysbiosis but also with altered SCFA production [33]. These two events are an excellent example of the preponderant protective role of the microbiota not only by its mere presence but further by its metabolites. 

## 7. Microbiome-based Therapeutics as a Potential Intervention for IBD and Comorbid MetS

Gut-derived metabolites, shaped by the microbial ecosystem residing in the gut, serve as a potential common origin connecting IBD, MetS and associated diseases like T2DM. When treating IBD, targeting comorbidities via shared pathways should be a consideration for therapeutic interventions. The microbiome-derived metabolites comprise the main focus here, and modulating the microbiome composition using various therapies including antibiotics might be a potential solution. However, as highlighted by Kostic and collaborators (2014), even when antibiotics aid in IBD remission (specifically for pouchitis), this is not a ‘one-size fits all’ approach, since it may backfire by promoting enrichment of harmful or antibiotic-resistant species, and more importantly it may affect metabolic signatures in still unknown ways. From this perspective, the addition of beneficial microorganisms through the development of microbiome-based interventions, like fecal transplantation and well rationalized and designed oral probiotics, is promising [256]. Whilst fecal transplants are actively being studied as a clinical option for the treatment of UC as well as obesity and T2DM, it is still controversial and challenging since identifying appropriate donors is difficult. On the other hand, probiotic therapy represents a widely accepted way to restore gut health [257]. Some of the benefits reported for probiotic intake rely on its ability to promote a direct increase in beneficial gut microbiota metabolites [257], whilst others promote the secretion of antimicrobial compounds [258], thus protecting against pathogens. Furthermore, certain probiotic strains can enhance or suppress [259,260] the immune response in favor of the host. Overall, however, a somewhat narrow range of probiotics are commercially available and viewed as a one-size fits all model. Despite the potential health benefits of probiotics, there are limitations. Although many probiotics have been isolated from stool samples of healthy individuals, their consumption does not result in stable gut colonization [261]. Colonization resistance is the mechanism by which the native microbiome protects itself from invasive microbes [262] or probiotics [263]. This resistance may be the primary reason why most probiotics cannot readily occupy space in the gut [264]. Indeed, currently available probiotics are not clinically effective for IBD [265,266] or produce conflicting results [267,268,269]. Similarly, while it has also been proposed that probiotics can be of benefit to reduce insulin resistance [270] and help weight control [271], this has not been clinically proven. A focus on probiotic benefits that are metabolite derived could also be effective, considering *Lactobacillus reuteri* has been reported to secrete AhR ligands and aid in the metabolism of SBA. For most of these protective effects to occur, probiotics must effectively colonize and persist in the gut [272], which may be unlikely given that gut inflammation is so pervasive in these conditions [185,213]. Given the prominent role the gut microbiota plays in IBD and insulin resistance, discovering new strains of beneficial bacteria targeting gut inflammation or genetically engineering probiotics as bugs as drugs, targeting common metabolites may revolutionize IBD treatments that decrease symptoms in the gut but also the associated metabolic diseases.

## 8. Conclusions

Classically, the relationship between MetS and IBD has not been investigated, as lack of proper gut function in IBD hampers nutrient absorption and, hence, presents an unlikely view of developing MetS, commonly associated with nutrient overexposure. This conundrum exists definitely among untreated/undiagnosed IBD patients that often present with a low BMI and, thus, might not present classic symptoms of MetS. However, as recognition of IBD has increased and most patients in North America are being treated, being underweight has become less of an issue. Similarly, as MetS moves away from chronic nutrient overexposure to inflammation as being the root cause, comorbid experiences with other inflammatory diseases like IBD are being increasingly recognized. In light of the aberrant microbiome being a key culprit between both diseases, it is time to perhaps consider these two very different diseases as being two sides of the same coin. The connections between IBD and MetS are multiple, and further studies directed towards understanding the role of microbial dysbiosis, one of the root causes of bacterial infiltration induced inflammation connecting IBD and MetS, are needed. So far, the dysbiotic gut appears to sculpt the inflammatory environment in the gut through decrease in the anti-inflammatory metabolites such as butyrate, IPA and SBA and constitute increase in the pro-inflammatory BCAAs and TMAO. As described in the text, this combination may have a powerful and detrimental effect on the gut barrier, extending the wave of adverse events to induce metabolic defects in the liver and pancreas, including increased liver lipogenesis and insulin impairment in the pancreas. These may not stop there, as both organs communicate back to the gut, setting detrimental loops for the host. Experimental data supporting this includes mice models where oral insults lead to gut and liver defects. In humans, these observations are increasing, and as omics studies continue to generate data, the connections between IBD and metabolic disturbances will be clearer and easier to address. Lastly, we would like to stress the burden and importance of IBD and MetS as life-long diseases. Although a long-term cure with no detrimental aftermath is the ultimate goal, no rationale advance can be developed until we include the microbiome and its metabolites as major players of our healthy or diseased status.

## Figures and Tables

**Table 1:** Comparison of metabolic disturbances in chemical and genetic derived colitis rodent models.

Model	Colitis Model	Evidence for m 0.75 Metabolic Defects	Trigger for Exaggerated Metabolic Defects
DSS	DSS exposure via drinking water; low doses (e.g., <3%) lead to mild symptoms and high doses (>3%) to acute disease. The ulceration is superficial, almost restricted to the colon [95].	DSS per se does not cause liver damage [96] but alters the systemic metabolism. LDL-C, ketone bodies and tryptophan are elevated, while glucose and amino acids are reduced in DSS-induced colitis model [83,96,97,98].	HFD (60% cocoa butter or D12492 diet) [78,84], choline deficient diet [99] or ApoE^−/−^ [85] model worsens pre-existing colitis scores, and further induced endotoxemia, glucose impairment, liver fibrosis and steatosis.
TNBS	TNBS causes transmural colitis, and it needs ethanol as vehicle for enema administrations, which also aid in disrupting intestinal barrier.	Cause liver damage [79,100,101], accompanied by intestinal inflammation and endotoxemia.	HFD (fat from lard) further worsens colitis scores [87,88], endotoxemia [86] and fat deposition in liver and colon [87].
IL-10^−/−^	Spontaneous development of chronic inflammation due to exacerbated Th1 and Th17 response in the absence of the anti-inflammatory IL-10 cytokine [80].	Decrease serum glucose [80] but increases in urine [102]. Increase serum VLDL [103].	APN^−/−^, IL10^−/−^ double knockout mice do not exaggerate the IL-10 deficiency induced colitis [80].
Mdr1a^−/−^	Spontaneous development of bowel inflammation due to absence of P-glycoprotein, associated [104].	Mdr1a^−/−^ mice are similar to their congenic background strain FVB [104] which are resistant to diet induced obesity.	HFD (30.5% fat from lard) exposure only worsen IBD disease score, without affecting liver or glycemic response [81].
Muc2^−/−^	Defective mucin secretion leads to spontaneous development of colitis [82].	Muc2^−/−^ mice show less body weight gain and impaired glucose homeostasis [82].	HFD (59% fat, mostly from lard) does not worsen glucose intolerance but induces the fat deposition in the liver [82].

APN: adiponectin; DSS: dextran sulfate sodium; ApoE: apolipoprotein E; HFD: high-fat diet; LDL-C: low-density lipoprotein cholesterol; Mdr: multidrug resistance protein; Muc2: mucin2; TNBS: 2,4,6-trinitrobenzene sulfonic acid.

**Table 2:** Clinical and rodent studies assessing the role of gut-derived metabolites commonly involved in the pathogenesis of inflammatory bowel disease and co-morbid metabolic defects.

Metabolites	Clinical Importance	Roles in IBD		Roles in Metabolic Disorders	
		Human	Rodent	Human	Rodent
BCAA (Leucine, Isoleucine, and Valine)	Maintain the protein synthesis and muscle growth [110].Ensure the intestinal integrity and immune response [111].	Increase the development and severity of pre-existing colitis [112]. Increase (high dose, >2.57%) intestinal immune response [112,113] by activating mTOR and NF-kB [114].	Worsen DSS-induced colitis following the diet containing animal-based protein [115]. May increase the severity of chemical-induced colitis through excessive activated colonic macrophages [116]. Promote systemic oxidative stress and the activation of inflammasome, leading to extensive intestinal inflammation [117].	Remodel lipid metabolism (increase LDL-C and triglycerides). Increase the risk and development of CVD [118,119], obesity [120,121,122], insulin resistance [122,123] and hepatic diseases [121,124].	Induce body weight gain, hyperglycemia, insulin resistance and accumulation of hepatic lipid droplets [125]. Impair insulin sensitivity, cardiac function (EF%) in mice received transverse aortic constriction [126].
Tryptophan metabolites	Provide indirect assistance on maintaining intestinal permeability and epithelial integrity [127].	Decrease in serum of patients with UC [128,129] and CD [128,130]. Stronger potential of tryptophan degradation in active IBD cases [128].	Attenuate severity of DSS-induced colitis by limiting the secretion of inflammatory markers [131,132]. Serve as a treatment (IPA) of active IBD remission in mice by enhancing anti-inflammatory responses [133]. Provide intestinal antifungal resistance by producing IL-22 [134].	Negatively correlates to insulin deficiency and glucose imbalance in diabetes patients [135]. Reduce serum tryptophan [136] and IPA [137] in patients with obesity and T2D.Improve overweight correlated inflammatory response [134].	Reduced body weight gain (IPA), improved glucose metabolism and insulin resistance in obese mice [138,139]. Ameliorate active colitis cases by enhancing T-cell dependent immunity and upregulating AhR [140]. Improve insulin sensitivity, glucose homeostasis and energy regulatory hormones (e.g.,: leptin and GLP-1) [141]. Protect against intestinal permeability and systemic immunity [142].
SCFA	Control systemic energy metabolism and regulate intestinal immune response [143].	Prevent incidence and development of IBD (butyrate and propionate) [144,145]. Increase risk of IBD (acetate and pyruvic) [146]. Enhance anti-inflammatory potential (butyrate), therefore improving the pre-existing IBD [147].	Improve IBD by suppressing T-cell mediated inflammatory responses (butyrate) [148]. Protect against colitis susceptibility and improve intestinal permeability in DSS-induced colitis model [149].	Provide protection on developing insulin resistance, obesity and diabetes (butyrate and propionate) [150]. Improve severity of obese, insulin resistance, diabetes and glucose homeostasis (propionate) [151,152]. Negatively correlates with hypercholesterolemia (butyrate) [153].	Protect against high fat-feeding induced liver steatosis and insulin resistance in mice and rats [154,155,156,157,158,159].
Bile acid	Maintain enterohepatic circulation, systemic energy homeostasis and the balance of gut bacterial community [160].	Decreased bile-acid transforming bacteria in IBD patients [161,162]. Negatively correlate with CD (SBA and conjugated bile acids), but not UC [1,4,5,163]. SBA and conjugated bile acids can be restored by applying anti-TNF-α treatment [164].	Protect against epithelial permeability and goblet cell loss by activating FXR-α [165,166]. Improve anti-inflammatory response by maintaining RORγ+ regulatory T cells in IBD mice [167].	Accumulate in patients (mostly primary bile acid) with liver dysfunction (hepatic steatosis, lobular and portal inflammation) [168] obesity [169] and diabetes [170], with interrupted bile acid negative feedback loops via FXR-α [143,160].	Improve hepatic glucose metabolism and insulin resistance by upregulating FXR-α [164]. Improve insulin resistance [171] and lipid metabolism [172] via anti-obesity receptor in mice model with metabolic stress.
TMA/TMAO	Classic risk factor on inducing chronic diseases [173].	Positively correlates with active UC and CD cases [174].	Indicate disrupted gut bacterial ecology by overexpressing choline associated catabolic enzymes [175]. Accelerate the progression of IBD in colitis mice models [175,176].	Indicate the risk, incidence [177,178,179] and mortality rate [180] of cardiovascular diseases Increase platelet responsiveness [174].Increase the incidence of insulin resistance [181] and T2D [178].	Increase aortic lesion, platelets responsiveness [165,167], and microbiota dependent atherosclerosis [176] in mice supplemented with TMAO. Increase inflammatory biomarkers (e.g., NF-kB) [59], hyperglycemia [182].Induce the formation of ox-LDL [165] and NAFLD through disrupted choline metabolism [183].
